# Pomegranate Peel Powder: In Vitro Efficacy and Application to Contaminated Liquid Foods

**DOI:** 10.3390/foods12061173

**Published:** 2023-03-10

**Authors:** Valentina Lacivita, Adriana Lordi, Tamara Posati, Roberto Zamboni, Matteo Alessandro Del Nobile, Amalia Conte

**Affiliations:** 1 Department of Agricultural Sciences, Food and the Environment, University of Foggia, Via Napoli, 71122 Foggia, Italy; 2 Consiglio Nazionale delle Ricerche, Istituto per la Sintesi Organica e la Fotoreattività (CNR-ISOF), Via Piero Gobetti 101, 40129 Bologna, Italy

**Keywords:** pomegranate peel, fruit by-products, soymilk, apple juice, natural additives

## Abstract

In this study the recycling of pomegranate peel powder (PPP) was proposed. In particular, the use of powder loaded in a silk fibroin polymeric matrix to create an active pad was tested. For the sake of comparison, the powder alone was also analysed. Both powder and active pad efficacy was assessed in two different food systems, soymilk (rich in proteins), preliminarily contaminated with *Pseudomonas* spp. and yeasts, and apple juice (rich in carbohydrates), preliminarily contaminated with *Alyciclobacillus acidoterrestris*. Three different concentrations of powder alone and powder in the pad were tested (5%, 7.5% and 10% *w*/*v*) in both types of beverages. To assess a possible dependence of the efficacy on the powder granulometry, different powder sizes were preliminarily analysed on *Pseudomonas* spp. and yeasts using an in vitro test. PPP was effective on both *Pseudomonas* spp. and yeasts. No significant differences appeared among the tested granulometries and therefore in the subsequent tests powder with an average diameter of 250 µm was used. Results recorded with soymilk and apple juice were different. When applied to the soymilk, the activity of PPP in the pad was less effective than that recorded when the powder was directly added to the beverage. With the two highest powder concentrations directly added to food, more than four log cycle reductions in *Pseudomonas* spp. and yeast cells were recorded, compared to soymilk without any powder. Compared to the control sample, all the soymilk samples either with PPP or with the active pad showed a delayed microbial and fungal growth. When applied to apple juice, both powder and pad were effective at completely inhibiting the proliferation of *A. acidoterrestris* (<10^2^ CFU/g).

## 1. Introduction

In the last years, food waste has become a global problem with serious implications on economic aspects and on the environment, and above all on climate, soil, water resources and biodiversity [1]. Therefore, waste reduction should become a priority. To date, reuse within the human food chain seems to be the best option [2]. Most food by-products come from fruit and vegetable processing in terms of seeds, peel, and leaves. These by-products, being rich in polyphenols and flavonoids, are considered natural antimicrobial and antioxidant agents [3], and therefore they are prized for their nutritional properties [4]. For this reason, by-products, or their extracts, can be recycled and reused as raw materials, to produce fortified foods [5,6] or to prolong fresh food shelf life [7,8,9,10].

Among the various by-products, pomegranate peels and their extracts gained great attention as they turned out to be powerful antimicrobial and antioxidant sources of compounds, such as tannins and anthocyanins [11]. The pomegranate fruit consists of 50% peel. A recent estimate considered the pomegranate peel world production to be about 1.9 million metric tons per year [12]. Due to the abundant amount, several uses of residues from processing of pomegranate were evaluated. As recently reviewed by El Barnossi et al. [12], these by-products may be used directly or after treatments as animal feed, bio-fertilizer, bio-adsorbent, production of biogas, bioethanol, biohydrogen and value-added products such as essential oil, enzymes, food, medical and cosmetic products. 

In the food sector, Incoronato et al. [13] designed a fortified pancake with all parts of pomegranate (juice and by-products), Bourekoua et al. [14] evaluated the effect of the addition of pomegranate seed powder on physical, sensorial and antioxidant properties of gluten-free bread. Another interesting case-study concerns the possibility of extending the shelf life of fresh fish burgers fortified with whole pomegranate juice, peel, and seeds [15]. The use of pomegranate peel powder up to 3% was also proven to be effective as a natural preservative in high quality beef sausages during storage at 4 °C [16].

From the literature, it emerges that the use of by-products as sources of bioactive compounds to develop films with antimicrobial and/or antioxidant properties is also very common [17,18]. For example, pomegranate components have been used for the development of active polymeric systems [19]. Mushtaq et al. [20] made a zein-based film by incorporating pomegranate peel extracts in the polymeric matrix. Bertolo et al. [21] functionalized chitosan/gelatin-based materials by pomegranate peel extract. Chitosan-based coatings with incorporated pomegranate peel extracts have been applied to rainbow trout [22] and Pacific white shrimp [23]. Giannelli et al. [24] recently developed an active pad based on silk fibroin and pomegranate peel powder, with the potential to be adopted for food packaging. It is abundantly recognized that for regulatory reasons, delivery systems of active compounds are advantageous. A sustained release of phenolics over time is observed, compared to dipping foods in phenolic solution or adding a phenolic solution to the food before packaging [25]. In addition, biopolymers from biowastes offer great opportunities to decrease the devastating overuse of plastic-based packaging [18]. Starting from results of Giannelli et al. [24], in the current study, the active pad made up of silk fibroin and pomegranate peel powder was applied to contaminated fresh liquid food. The active pad was developed by considering that silk fibroin acted as a reservoir of antimicrobial phenolics contained in the pomegranate peel, which reached the food mainly through diffusion. In our active pad it was expected that release rate of peel phenolics over time would be adequate to maintain a sufficient concentration to inhibit growth of unwanted microorganisms and yeasts in two types of perishable food beverages. 

Milk-based drinks and fresh fruit juice are beverages prone to microbial deterioration during storage, as well as to chemical, physical and sensorial changes [26,27,28]. Alternative preservation techniques to thermal processing have been proposed for fresh beverages to satisfy consumer demand for more fresh-tasting juice; however, these solutions are still limited and require further optimization to find a large-scale application [29]. For example, juices were treated by high pressure [30,31], also combined with ultrasound and pulsed electric field [32], by microwave heating and thermo-sonication [29,33,34], by cold atmospheric pressure plasma [35] and by carbonation [36]. 

In view of the world production of a plentiful amount of waste generated by pomegranate fruit and considering that these wastes present risks to the environment and human health and also taking into account the increase in their economic benefits, the pomegranate by-product valorisation through an active pad development could be very promising. Therefore, from the perspective of taking advantage of by-products and reduction of waste, the aim of this study was to develop an active pad loaded with pomegranate peel powder. For comparison, the peel powder alone was also tested. A preliminary in vitro test was made to assess differences among different powder granulometries. Once the proper powder size was selected, three concentrations of pomegranate peel powder, alone and in the pad were, respectively, tested against *Pseudomonas* spp. and yeasts, previously inoculated in soymilk, and against *A. acidoterrestris* previously inoculated in apple juice. The ability of the powder alone and of the active pad in improving the microbial stability of the two previously inoculated fresh food beverages was assessed during an appropriate storage period.

## 2. Materials and Methods

### 2.1. Pomegranate Peel Powder Preparation and In Vitro Efficacy

The pomegranate peel powder (PPP) was obtained according to the study of Giannelli et al. [24] from the pomegranate fruit of the Wonderful variety, kindly supplied by A.P.O. Association (Foggia, Italy). Briefly, pomegranates were carefully washed, rinsed and cut to separate the peel from the arils. The by-products were dried in a food dehydrator (at 38 °C for 48 h) up to a final humidity of 8.77%. After drying, the by-products were ground into a powder in a lab blender and then sieved. Different granulometries were obtained, 100, 150, 250, 400 and 500 µm, using correct sieves (ENCO srl, Spinea, Italy). The PPP with five different sizes, was tested against *Pseudomonas* spp. and yeasts, as typical food spoilage organisms. In particular, *Pseudomonas putida* and *Pseudomonas fluorescens* isolated from spoiled mozzarella cheese, and yeasts isolated from red grape marc, were used. The two strains of *Pseudomonas* spp. were maintained in Plate Count broth (PC, Oxoid, Milan, Italy) at −20 °C with the addition of 30% of glycerol as stock cultures. Prior to the antimicrobial tests, exponentially growing cultures were obtained by allowing each strain to grow in appropriate broth at 25 °C for 24 h. Then, a cocktail of the two strains was prepared by mixing 1% of each culture. The cocktail of *Pseudomonas* spp. and yeasts at a concentration of 10^3^ CFU/mL were both prepared by diluting growing cultures with sterile saline solution (9 g/L NaCl). For testing the powder efficacy, each granulometry of PPP (5% *w*/*w*) was placed in 20 tubes containing 10 g of PC broth (10 tubes for *Pseudomonas* spp. and 10 for yeasts). All tubes were incubated at 25 °C for 72 h. At different incubation times (4, 24, 48 and 72 h) aliquots of 1 mL were taken from each tube for microbiological analyses carried out by plate count (incubation at 25° C for 48 h). All analyses were performed twice on two different samples. In all the samples the measurement of pH, conducted in triplicate, was performed on the first homogenized dilution of the samples, by a pH meter (Crison, Barcelona, Spain) with an accuracy of 0.01 pH, previously calibrated with 4.01 and 7.00 pH buffer solutions.

### 2.2. Pad Development

Pads based on silk fibroin, with and without pomegranate peel powder (PPP), were prepared according to the study of Giannelli et al. [24]. To extract fibroin, *Bombyx mori* silkworm white cocoons (CREA, Padua, Italy) were used. The silk fibroin extraction was carried out according to details also reported in the abovementioned article of Giannelli et al. [24]. The active pad, at a ratio 30:70 by mass (Silk fibroin:PPP), was prepared by blending a 6% *w*/*v* of water silk fibroin solution containing 20% *w*/*w* of glycerol vs. silk fibroin to induce water insolubility [37], with a suitable amount of pomegranate powder. The active pad was obtained by using a support of polydimethylsiloxane (5 × 4 cm) as substrate. Five mL of the obtained blend were drop-casted, left to dry at room temperature, and peeled off from the substrate. As control, pads of the same dimensions without any powder were also prepared.

### 2.3. Scanning Electron Microscopy (SEM)

SEM analysis was performed with a Zeiss EVO LS 10 LaB6 scanning electron microscope. (U.S. Headquarters, New York, NY, USA). The PPP (2–3 mg) and the obtained silk fibroin-PPP pad (about 0.5 cm × 0.5 cm) samples were deposited on standard aluminium substrate and observed at an acceleration voltage of 5 kV and a working distance of 5 mm.

### 2.4. PPP and Active Pad Efficacy in Previously Inoculated Soy Milk and Apple Juice

The PPP, the control pad and the active pads were tested in two different liquid foods: the soymilk, which is rich in proteins, and the apple juice, which is rich in carbohydrates. The soymilk and apple juice compositions reported on the packaging of each product are listed in Table 1.

To simulate contaminated soy milk, the samples were inoculated with *Pseudomonas* spp. (mix of *P. putida* and *P. fluorescens*) and yeasts. The inoculation (10^3^ CFU/mL) was obtained by diluting growing cultures of *Pseudomonas* spp. and yeasts in soymilk. Different concentrations of PPP (granulometry 250 µm) were tested, specifically, 5%, 7.5% and 10% (*w*/*v*). The same PPP concentrations in the liquid food were used when the active pad was tested. Therefore, the grams of pad necessary to reach the same powder amount in the milk were calculated keeping in mind that the concentration of PPP in the active pad is 30:70 by mass (silk fibroin:PPP). Specifically, 0.8 g of pad corresponded to 5% of PPP in the liquid food, 1.2 g corresponded to 7.5%, whereas 1.6 g corresponded to 10%. In each tube, soymilk (10 g), with a proper amount of either PPP or active pad, were mixed and then stored at 4 °C. For the control sample, the tubes were inoculated and stored without adding any powder/pad. At different storage times aliquots of 1 mL were taken from each tube for microbiological analyses (plate count technique at 25 °C for 48 h). All analyses were performed twice on two different samples.

**Figure 1:**
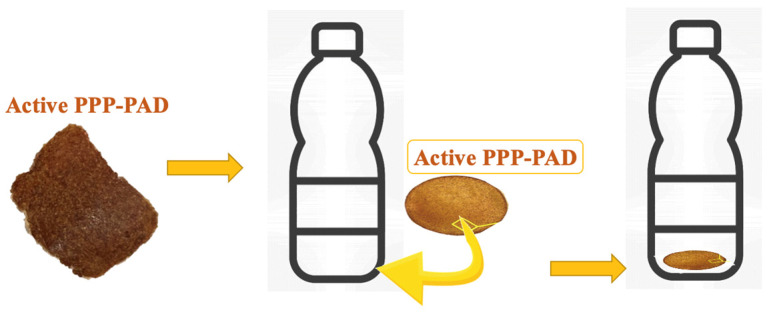
Pad developed in the current study and its future application in a beverage bottle.

The PPP and the active pad efficacy were also tested in apple juice contaminated by *A. acidoterrestris*. The *A. acidoterrestris* (DSM 3922) was provided by DSMZ (Germany). It was revitalized in Malt Extract Broth (MEB, Oxoid, Milan, Italy) at 44 °C for 48 h. The inoculum in apple juice was prepared by diluting the overnight culture to approximately 10^3^ CFU/mL. In each inoculated tube containing 10 g apple juice, 5%, 7.5% and 10% (*w*/*v*) of PPP (granulometry 250 µm) and 0.8 g, 1.2 g or 1.6 g of the active pad were added, respectively. For the control samples, some tubes were inoculated and stored without adding any powder/pad. All the apple juice samples were stored at 37 °C for two weeks. During storage, an aliquot of 1 mL was taken from each apple juice, diluted and then 0.1 mL was spread on Malt Extract Agar (MEA, Oxoid, Milan, Italy). For *A. acidoterrestris* count, the plates were incubated at 44 °C for 48 h. The pad in both soymilk and apple juice was placed on the bottom of the container and the liquid product was then added. The pad did not float in the liquid food, it did not disintegrate or break. In Figure 1 the original pad developed in the current study and its future potential application in beverage bottle is represented.

In the broths and in all the soymilk and apple juice samples the measurement of pH was carried out as reported in the previous Section 2.1.

### 2.5. Data Elaboration and Statistical Analyses

All the experimental data were compared by one way ANOVA and Duncan’s multiple range test with the option of homogeneous groups to highlight significant differences among them (*p* < 0.05). STATISTICA 7.1 for Windows (StatSoft, Inc., Tulsa, OK, USA) was the software adopted.

## 3. Results and Discussion

### 3.1. In Vitro Test: Influence of Granulometry on PPP Efficacy

**Figure 2:**
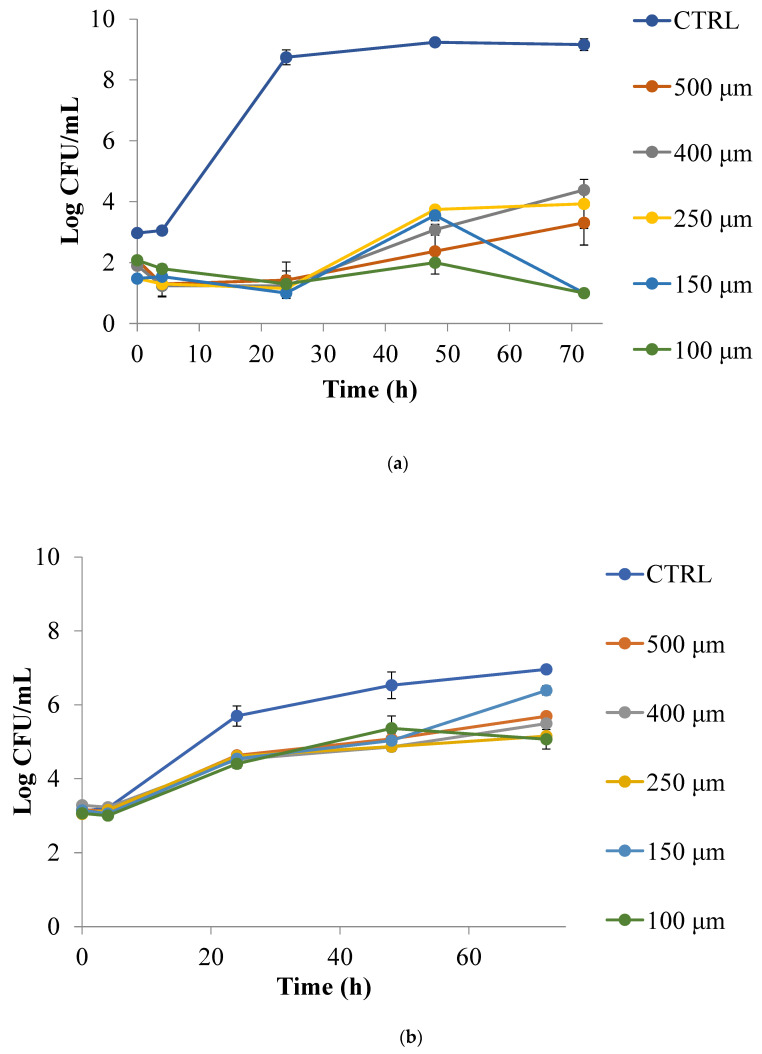
*Pseudomonas* spp. (**a**) and yeasts (**b**) in PC broth with (5%) and without PPP with granulometry from 500 to 100 μm.

PPP at different sizes was preliminarily tested on *Pseudomonas* spp. and yeasts under in vitro conditions to assess if any dependence between efficacy and powder granulometry existed. Results recorded with powder in contact with the selected microbial and fungal species are reported in Figure 2a,b. As can be seen, Figure 2a highlights a great efficacy of PPP on the two selected *Pseudomonas* strains. In fact, in the control sample the microorganisms grew very rapidly and within one day reached 10^8^ CFU/mL, whereas in the samples with PPP the microbial proliferation was substantially inhibited and remained below 10^4^ CFU/mL during the entire observation period. As abundantly recognized, foods contaminated by *Pseudomonas* spp. become unacceptable when the viable cell concentration exceeds 10^6^ CFU/mL, and therefore data recorded assessed an important efficacy of PPP against this spoilage group. Any significant differences among samples (*p* < 0.05) were observed. In Figure 2b the trend of yeasts is reported. In this case the control sample reached the undesired yeast concentration of 10^5^ CFU/mL within about the first 20 h of monitoring, whereas it took about the double time to reach the same fungal concentration for all the samples enriched with the powder. As can be inferred from the data shown in the figure, no significant differences among sizes can be highlighted (*p* < 0.05). Considering the recognized properties of peel fruit on yeasts and various gram-negative and gram-positive bacteria yet reported in the literature, our results are in line with other scientific data [38,39,40,41]. The peel effects can be ascribed to phenolic compounds, especially anthocyanins and tannins [38]. In a previous study, we also found high contents of polyphenols and flavonoids in the same variety of pomegranate peel [13]. Recently, Xiang et al. [39] reviewed the bioactivity of pomegranate peel extract, highlighting that it could contain up to more than 20 polyphenol compounds with a variety of biological activities, such as antioxidative, antitumour, anti-inflammatory, neuroprotection, anti-viral, and anti-bacterial. 

The most accredited mechanism of action of pomegranate peel is due to the position of the hydroxyl groups (OH) in the aromatic ring of polyphenols. These groups interact with microbial cell membranes, provoking loss of cellular components and damaging metabolic processes [38,42].

As regards pH, Table 2 and Table 3 report data of broths inoculated with *Pseudomonas* spp. and yeasts, respectively. Apart from some significant differences among samples (*p* < 0.05), the most striking feature of the data is the fact that pH of the control samples in both cases ranged between 6 and 7, whereas for all the samples with PPP pH values around 4 were measured. This finding is not surprising considering that pH of PPP is less than 5 [12]. The same pH reduction was also found when pomegranate peel was added to brine of fresh-cut fruit, to a pancake formulation and to a fish-based burger [13,15,43]. Considering that low pH generally favours yeast growth [44], the effects of PPP recorded in the current study against yeasts is very promising.

Our positive preliminary results, in line with other findings from the literature, gave us the possibility to select PPP at a size of 250 μm for the subsequent experiments, because no significant differences were recorded among the various sizes (*p* > 0.05) and because 250 μm was one of the most abundant amounts of powder recorded during sifting. 

### 3.2. SEM Images of Pad

**Figure 3:**
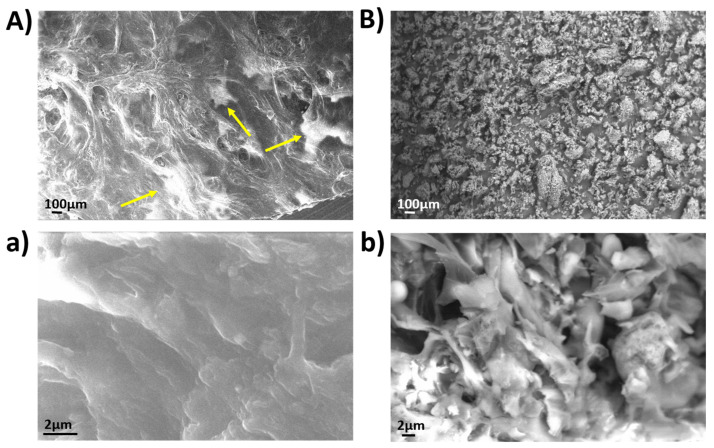
SEM images of the silk fibroin-based pad with PPP at different magnifications (**A**,**a**). SEM images of PPP at different magnifications (**B**,**b**).

The surface morphology of developed silk fibroin-PPP pad was investigated by SEM and compared with that of pristine PPP (Figure 3). As shown in Figure 3, the PPP sample was characterized by the presence of isolated grains (B and b), while the pad showed a more compact and continuous structure (A and a), due to the presence of silk fibroin protein that, as previously demonstrated in the work of Giannelli et al. [24], acts as glue for pomegranate powder particles. Moreover, in detail, no cracks in the pad surface areas were detected (A and a), but many white dots probably related to the insoluble aggregates of pomegranate peel particles embedded in the silk fibroin matrix were observed. This kind of morphology was already observed for other fruit peels incorporated in polymeric matrices as chitosan or gelatin [45]. 

### 3.3. PPP Efficacy in Previously Inoculated Soymilk and Apple Juice

**Figure 4:**
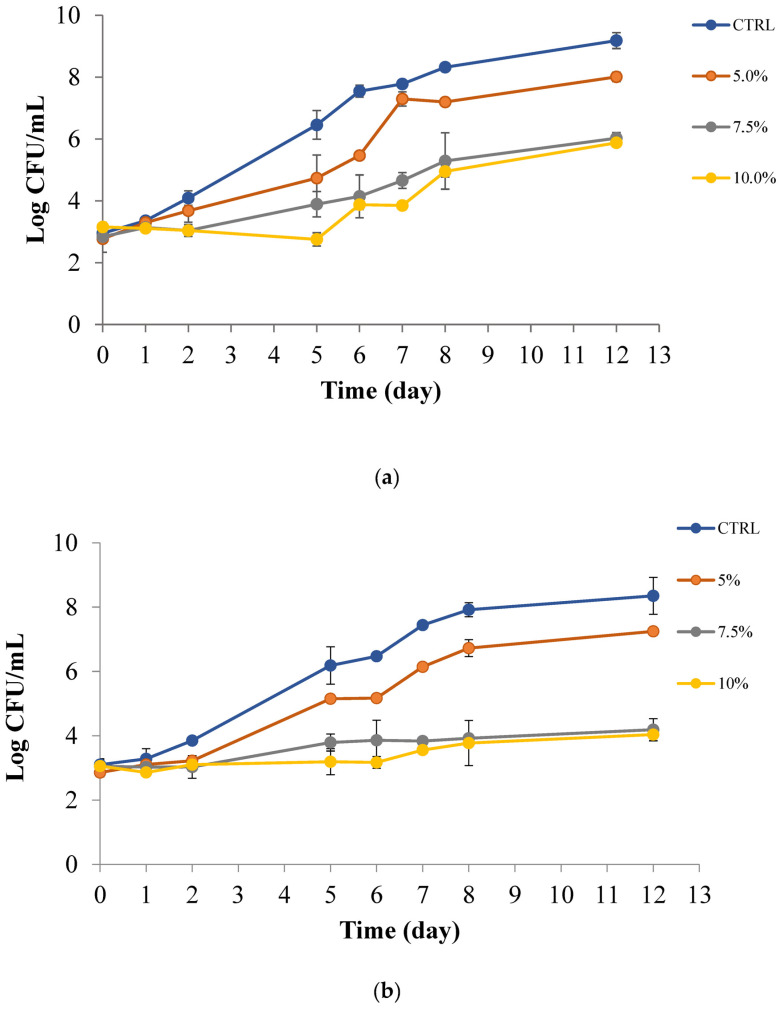
*Pseudomonas* spp. (**a**) and yeasts (**b**) in soymilk with PPP at 0, 5, 7.5 and 10% (*w*/*v*).

Data reported in Figure 4a,b show the trend of PPP efficacy in the soymilk inoculated with *Pseudomonas* spp. and yeasts, respectively, during about two weeks of storage under refrigerated temperature. As can be observed, Figure 4a highlights a control sample where *Pseudomonas* spp. rapidly grew and reached a high concentration within a few days. Samples of soymilk containing the fruit peel presented delayed growth, with lag phases that are more marked as the concentration of peel increased. The maximum viable cell concentration in the stationary phase was also different among samples. In fact, the sample with 5% *w*/*v* PPP reached about 10^7^ CFU/mL at the end of the observation period, whereas the viable cell concentration of the two samples with 7.5 and 10% *w*/*v* PPP remained around 10^5^ CFU/mL and therefore never reached completely unacceptable levels of *Pseudomonas* spp. proliferation. A similar situation can be observed for yeasts. As can be seen in Figure 4b, the control soymilk reached undesired fungal proliferation levels above 10^5^ CFU/mL within 4 days of storage. Patrignani et al. [44] considered a concentration equal to six log CFU/mL as the threshold for yeast acceptability in fruit juice. On the contrary, when added at 5% *w*/*v*, the PPP delayed yeast growth in soymilk and when added at 7.5 or 10% *w*/*v* it completely inhibited fungal proliferation. Therefore, results in the inoculated soymilk enriched with PPP demonstrate that the peel could be advantageously used in the beverage to control undesired spoilage.

Despite the effects recorded with PPP tested in the liquid foods, some differences can be seen in results of the in vitro test. In particular, PPP tested in the PC broth was more effective than in the inoculated soymilk. This experimental evidence can be ascribed to more than one reason: the binding of the active compounds of peel to food components, the protection of microorganisms in the real food matrix and the physiological state of microorganisms under refrigeration conditions, compared to temperatures optimal for microbial growth [46]. As regards interactions, many authors studied phenols and food constituents and observed that higher concentrations than the in vitro bactericidal concentration were necessary to get a bacteriostatic effect in food models. Literature data assessed that the loss of susceptibility of most fungi and bacteria is due to interactions of active phenolics with main food constituents, protein, and fat, at the expense of their interactions with unwanted microbial or fungal cells [47,48]. It is also true that unwanted microorganisms are protected by a “layer” of food constituents limiting the direct contact of antimicrobial phenolics with their cell envelope [49,50].

The pH values are in line with data recorded in the in vitro test, with control samples ranging between 6.4 and 6.8 and active samples ranging between 4.4 and 4.8 during the entire storage period.

**Figure 5:**
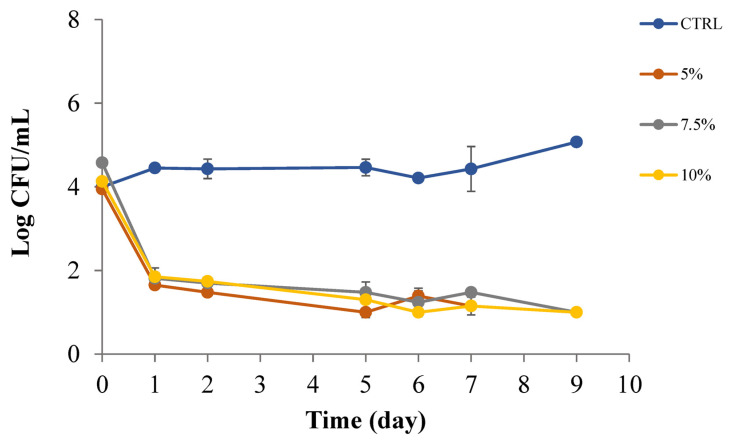
*A. acidoterrestris* in apple juice with PPP at 0, 5, 7.5 and 10% (*w*/*v*).

Data in Figure 5 report results of *A. acidoterrestris* viable cell concentration evolution in samples of apple juice, with and without PPP. This figure clearly shows the great effect of PPP on the spoiling *A. acidoterrestris* and highlights that even the smallest concentration of PPP (5% *w*/*v*) was enough to completely provoke the microbial cell death. The initial concentration of the microorganism in the juice samples was around 10^4^ CFU/mL, in the control juice the concentration slightly increased, while in the three juices with PPP it rapidly declined under any microbial detection level. No differences appeared among the three active samples (*p* > 0.05). Thus, PPP at tested concentrations is effective at controlling proliferation of cells of *A. acidoterrestris* in apple juice. 

The great efficacy of PPP in the juice compared to the effects recorded by using the same peel concentration in the inoculated soymilk could be ascribed to different food component interactions. In fact, studies regarding the effect of proteins on the antimicrobial activity of phenolics exist and demonstrate that the antibacterial activity reduction in phenolics in the presence of proteins is correlated with their affinity for proteins [25], which in our case were abundant in the soymilk and absent in the apple juice. Without any doubt, polyphenols also interact with carbohydrates that mainly characterize our apple juice but the studies on these types of interactions are scarce compared to those related to proteins, and therefore, in the absence of such data, the efficiency of PPP in preserving the apple juice can hardly be further discussed [46]. 

Table 4 reports data of pH in apple juice. As can be seen, pH was around 3.5 in all the samples and for the entire storage period, regardless of the PPP addition. This is because apple juice is an acidic food matrix, and the addition of PPP did not provoke any further pH reduction. 

### 3.4. Active Pad Efficacy in Previously Inoculated Soymilk and Apple Juice

**Figure 6:**
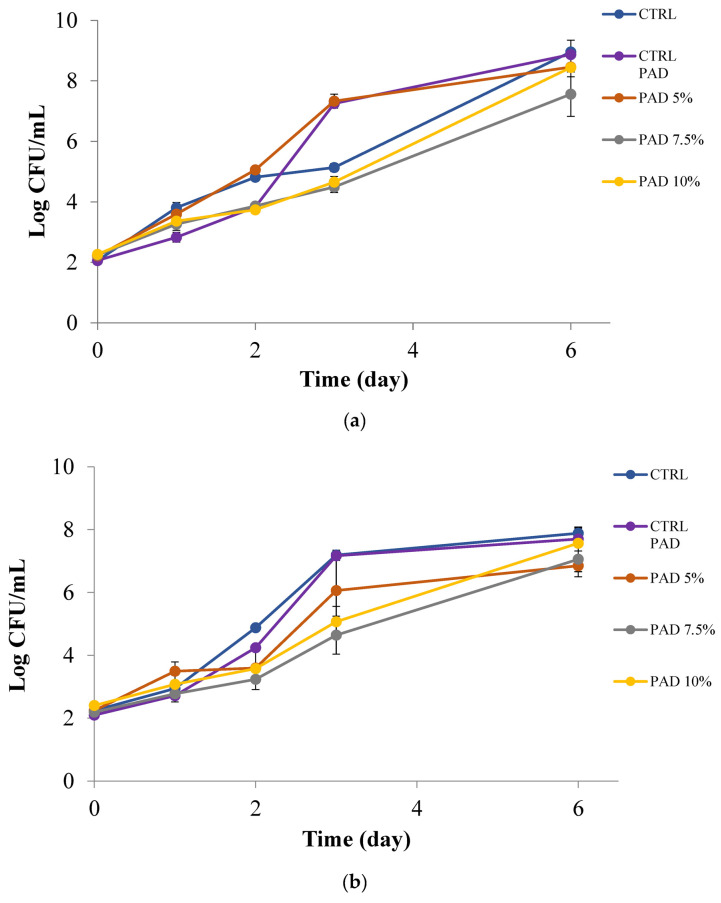
*Pseudomonas* spp. (**a**) and yeasts (**b**) in soymilk with and without pads at 0, 5, 7.5 and 10% (*w*/*v*).

**Figure 7:**
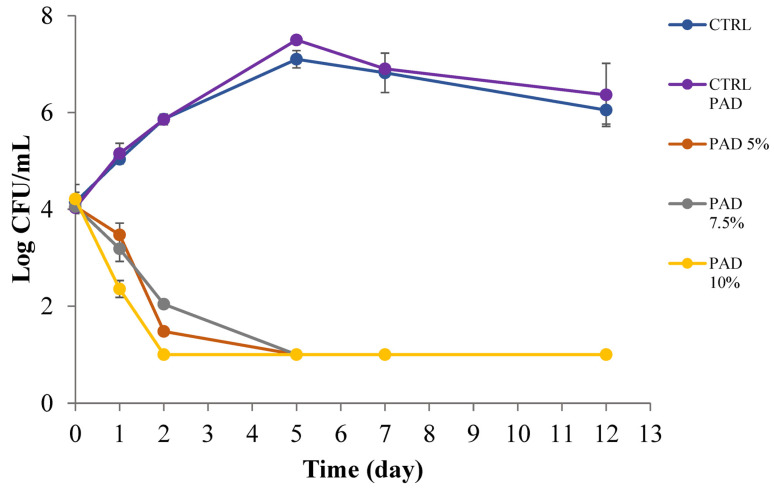
*A. acidoterrestris* in apple juice with and without pads at 0, 5, 7.5 and 10% (*w*/*v*).

Figure 6a,b shows the trends of microbial and fungal proliferation in the soy-based beverage, while Figure 7 highlights the evolution of *A. acidoterrestris* in apple juice. As can be seen in Figure 6, the microbial and fungal proliferations were not completely affected by the presence of the active pad, even though a certain inhibition can be observed with the two highest PPP concentrations in the polymeric matrix. In fact, looking at Figure 6a it is possible to infer that both the control samples and the soymilk with the active pad 5% *w*/*v* PPP presents similar behaviour to *Pseudomonas* spp. which reached undesired levels of 10^6^ CFU/mL after 2.5 and 3.5 days, respectively. In the two samples with 7.5 and 10% *w*/*v* PPP, the same undesired microbial growth was reached after more than 4.5 days, thus suggesting that higher concentrations than 5% *w*/*v* were suitable to release enough active compounds in the beverage to slow down microbial proliferation. A similar trend can be inferred in Figure 6b for yeasts. The two control samples and the active pad with the lowest PPP concentration became unacceptable for yeast growth within 3 days of storage at 4 °C, whereas the other two active soymilks remained acceptable for more time, about 4 days. These antimicrobial and antifungal effects confirmed data in the literature dealing with other bio-based polymeric matrices containing pomegranate peel or peel extracts, tested against various spoilage and pathogenic strains [51,52,53,54]. Kharchoufi et al. [55] showed good bacteriostatic effect by using different concentrations of PPP extract in chitosan film. These authors demonstrated, by an antifungal assay, that the bioactive coatings with the addition of the peel extract, significantly inhibited the growth of *Penicillium digitatum* by producing an inhibition halo around the experimental bioactive film disks. Direct comparisons between our developed active pad in soymilk with other delivery systems also based on pomegranate peel can be hard to extrapolate due to different polymeric matrixes used, different concentrations of peel powder tested, and different food items placed in contact [56]. In addition, it is worth considering that the amount of antimicrobial phenolics released is controlled by their partition equilibrium between the reservoir material and the food matrix in direct contact, and by kinetics of migration of phenolics in food contact material, and in food, respectively [46].

Compared to the powder itself, the pad was less effective which can be explained by more than one reason. The process of producing the active pad may have reduced the concentration of the antimicrobial phenolics present in the powder and consequently in the active pad. The release of the antimicrobial phenolics is hindered by the presence of the polymeric matrix, therefore, active compounds release rate from the active pad is slowed down compared to the powder itself. Last but not least, part of the antimicrobial phenolics could be absorbed by the polymeric matrix, therefore, if compared to the powder itself the amount of antimicrobial phenolics in the liquid food is diminished. 

The pH values of control soymilk and of soymilk with the control pad slightly ranged around 7 during the entire monitoring period. On the other hand, lower pH values around 5.9 were recorded in the samples when the respective three active pads were added to the beverage. Considering the low pH of pomegranate peel [12], and its release from the polymeric matrix, it was not surprising that a pH reduction (from about 5.9 to about 4.9) over time was recorded in the liquid food loaded with all the three pads.

As regards the behaviour of *A. acidoterrestris* in apple juice reported in Figure 7, a great inhibition effect of every active pad was recorded, as occurred with the simple PPP reported in Figure 5. In this case it is possible to observe a starting viable cell concentration around 10^4^ CFU/mL and two completely different microbial evolutions trends. In fact, the microbial cells proliferated in the two control juice samples but had a decreasing trend in the three samples containing the active pad, where the growth was completely inhibited. All the considerations reported in the previous paragraph dealing with the active pad efficacy in soymilk, are still valid in apple juice but we observed a more marked effect on *A. acidoterrestris* because the biomolecules’ concentration in this case was enough to inhibit the bacteria and therefore powder itself, or powder in the pad, recorded the same antimicrobial activity.

The values of pH in all the juice samples for the entire observation period were 3.5, as reported in Table 4 for the samples with powder. 

## 4. Conclusions

In this study pomegranate peel powder was tested against food spoiling microbial and fungal species. The powder itself, and its inclusion in a bio-based polymeric matrix were both considered. In vitro and in situ tests were carried out. Data recorded in vitro proved the effects of PPP on *Pseudomonas* spp. and yeasts, regardless of the powder granulometry. When the powder was tested in soymilk, a certain reduction in the efficacy was recorded, compared to the in vitro tests. This is most probably due to possible interactions between phenolic compounds and food components, in particular proteins. When the powder was tested against *A. acidoterrestris* in apple juice, a complete inhibition was found, probably due to the different food compositions of juice compared to soymilk, the former being constituted solely of carbohydrates. The same PPP concentrations were also tested in silk fibroin-based pads, also using soymilk and apple juice as liquid food systems. In this case also, the results were more interesting for the apple juice because the growth of the *A. acidoterrestris* was completely inhibited by the presence of the active pad, regardless of the PPP concentration. In the soymilk, only the two highest concentrations in the active pad exerted a certain bacteriostatic effect on microbial and fungal proliferation. These preliminary findings gave us interesting information on the powder alone, and on the delivery system based on silk fibroin and pomegranate peel applied to contaminated foods. Considering the obvious advantages in using bio-based polymeric systems that release active compounds, compared to direct addition of these molecules to food, further research could still be carried out to explore concentrations of PPP in the pad able to better preserve food from spoilage. In addition, the interesting experimental evidence recorded with the active pad in apple juice against *A. acidoterrestris* suggested that PPP in the polymeric matrix could be further reduced below 5% *w*/*v*, without affecting the antimicrobial efficacy. Considering that pads containing pomegranate by-products could better prevent sensory alterations in the food product, that are inevitable when the peel is directly added to the food matrix, pad optimization in terms of bacterial and fungal efficacy, would also require study of the preservation of food sensory properties. 

## Figures and Tables

**Table 1:** Composition of soy milk and apple juice.

Food Matrix	Soy Milk	Apple Juice
Fat	1.7 g/100 mL	-
Carbohydrates	2.9 g/100 mL	11 g/100 mL
Proteins	3 g/100 mL	-
Fibres	0.2 g/100 mL	-
Salt	0.10 g/100 mL	-
Vitamin B2	0.21 mg/100 mL	-
Vitamin B12	0.38 g/100 mL	-
Vitamin D	1.5 µg/100 mL	-
Calcium	120 µg/100 mL	-

**Table 2:** Values of pH for broths inoculated with *Pseudomonas* spp., with and without PPP.

Time (h)	CTRL	500 μm	400 μm	250 μm	150 μm	100 μm
0	6.89 ± 0.01 ^d^_E_	3.98 ± 0.00 ^a^_B_	4.02 ± 0.01 ^a,b^_C_	3.89 ± 0.02 ^a^_B_	3.94 ± 0.01 ^a^_A_	4.07 ± 0.01 ^c^_D_
4	7.01 ± 0.04 ^d^_E_	4.19 ± 0.01 ^d^_D_	4.07 ± 0.01 ^c^_C_	4.04 ± 0.01 ^b^_B,C_	3.97 ± 0.01 ^a^_B_	3.81 ± 0.12 ^a^_A_
24	6.03 ± 0.02 ^a^_D_	4.06 ± 0.03 ^b,c^_C_	4.00 ± 0.02 ^a^_B_	4.02 ± 0.01 ^b^_B_	3.94 ± 0.01 ^a^_A_	3.96 ± 0.02 ^b^_A_
48	6.41 ± 0.04 ^b^_D_	4.04 ± 0.01 ^b^_C_	4.04 ± 0.01 ^b^_C_	4.04 ± 0.00 ^b^_C_	3.82 ± 0.03 ^a^_A_	3.92 ± 0.00 ^b^_B_
72	6.56 ± 0.05 ^c^_B_	4.08 ± 0.02 ^c^_A_	4.10 ± 0.02 ^d^_A_	4.08 ± 0.03 ^c^_A_	3.74 ± 0.45 ^a^_A_	3.97 ± 0.01 ^b^_A_

Data are reported as mean ± SD. Data in the same column with different superscript letters are statistically different (*p* < 0.05). Data in the row with different subscript letters are statistically different (*p* < 0.05).

**Table 3:** Values of pH for broths inoculated with yeasts, with and without PPP.

Time (h)	CTRL	500 μm	400 μm	250 μm	150 μm	100 μm
0	6.97 ± 0.00 ^d^_D_	4.13 ± 0.01 ^c^_C_	4.09 ± 0.00 ^c^_B_	4.10 ± 0.00 ^b^_B_	4.05 ± 0.00 ^c^_A_	4.04 ± 0.01 ^c^_A_
4	6.85 ± 0.02 ^c^_D_	4.05 ± 0.00 ^b^_C_	4.06 ± 0.02 ^b,c^_C_	4.07 ± 0.01 ^a,b^_C_	3.87 ± 0.03 ^a^_B_	3.83 ± 0.02 ^a^_A_
24	6.82 ± 0.11 ^c^_C_	4.12 ± 0.00 ^c^_B_	4.16 ± 0.01 ^d^_B_	4.19 ± 0.07 ^c^_B_	3.98 ± 0.02 ^b^_A_	3.88 ± 0.05 ^a^_A_
48	6.48 ± 0.02 ^b^_E_	4.13 ± 0.01 ^c^_D_	3.94 ± 0.01 ^a^_B_	4.01 ± 0.01 ^a^_C_	3.99 ± 0.01 ^b^_C_	3.86 ± 0.02 ^a^_A_
72	6.25 ± 0.02 ^a^_C_	4.03 ± 0.02 ^a^_B_	4.04 ± 0.03 ^b^_B_	4.05 ± 0.01 ^a,b^_B_	3.97 ± 0.01 ^b^_A_	3.99 ± 0.01 ^b^_A_

Data are reported as mean ± SD. Data in the same column with different superscript letters are statistically different (*p* < 0.05). Data in the row with different subscript letters are statistically different (*p* < 0.05).

**Table 4:** Values of pH for apple juice inoculated with *A. acidoterrestris*, with and without PPP.

Time (Day)	CTRL	5%	7.5%	10%
0	3.52 ± 0.01 ^b^_A_	3.54 ± 0.01 ^c^_A_	3.52 ± 0.01 ^b^_A_	3.54 ± 0.01 ^c^_A_
1	3.46 ± 0.02 ^a^_A_	3.46 ± 0.02 ^b^_A_	3.48 ± 0.01 ^c^_A,B_	3.50 ± 0.00 ^b^_B_
2	3.55 ± 0.01 ^c^_A_	3.58 ± 0.01 ^d^_B_	3.56 ± 0.01 ^d^_A_	3.55 ± 0.00 ^c^_A_
5	3.46 ± 0.01 ^a^_A_	3.53 ± 0.00 ^c^_C_	3.52 ± 0.01 ^b^_B,C_	3.51 ± 0.01 ^b^_B_
6	3.45 ± 0.00 ^a^_A,B_	3.46 ± 0.01 ^b^_B_	3.44 ± 0.01 ^a^_A_	3.46 ± 0.01 ^a^_B_
7	3.51 ± 0.01 ^b^_C_	3.42 ± 0.02 ^a^_A_	3.44 ± 0.01 ^a^_A,B_	3.46 ± 0.01 ^a^_B_
9	3.51 ± 0.01 ^b^_B_	3.41 ± 0.01 ^a^_A_	3.42 ± 0.02 ^a^_A_	3.44 ± 0.02 ^a^_A_

Data are reported as mean ± SD. Data in the same column with different superscript letters are statistically different (*p* < 0.05). Data in the row with different subscript letters are statistically different (*p* < 0.05).

## Data Availability

Data will be made available on request.
